# Metabolomics Reveals the Effects of Nitrogen/Phosphorus/Potassium (NPK) Fertilizer Levels on Cucumber Fruit Raised in Different Nutrient Soils

**DOI:** 10.3390/metabo14020102

**Published:** 2024-02-01

**Authors:** Na-Rae Lee, Yangmin X. Kim, Yerim Lee, Chanwook Lee, Yosung Song, Hyejin Park, Choong Hwan Lee, Yejin Lee

**Affiliations:** 1 Research Institute for Bioactive-Metabolome Network, Konkuk University, Seoul 05029, Republic of Korea; nrlee3690@konkuk.ac.kr; 2 Soil and Fertilizer Division, National Institute of Agricultural Sciences, Rural Development Administration, Wanju 55365, Republic of Korea; yangmink@korea.kr (Y.X.K.); cksdnr0123@korea.kr (C.L.); sys531215@korea.kr (Y.S.); hjin11@korea.kr (H.P.); 3 Highland Agriculture Research Institute, National Institute of Crop Science, Rural Development Administration, Pyeongchang 25342, Republic of Korea; 4 Department of Bioscience and Biotechnology, Konkuk University, Seoul 05029, Republic of Korea; yerim333@konkuk.ac.kr

**Keywords:** mass spectrometry, cucumber metabolome, soil condition, fertilizer, pathway analysis

## Abstract

Fertilizers are widely used to improve the quality of fruits and vegetables. However, the overuse of fertilizers has become an issue because it causes environmental problems and negatively affects productivity and fruit quality. In this study, we examined the effects of nitrogen, phosphorus, and potassium (NPK) fertilizer levels on the metabolism of cucumber fruit in low- and high-nutrient soils using mass-spectrometry-based metabolomics approaches. Cucumber metabolite content was notably different depending on the initial soil nutrient status. Most amino acids and phenylpropanoids were abundant in the cucumbers raised in low-nutrient soil, whereas organic acids, some amino acids (aspartate, glutamate, and ornithine), and carbohydrates were comparatively higher in fruits from high-nutrient soil. The fertilizer supply resulted in an alteration in the metabolite profile, while no change in fruit yield was observed in either low- or high-nutrient soils. Fertilizer treatment perturbed the metabolite contents in cucumbers from low-nutrient soil. In contrast, treatment with higher concentrations of fertilizer in high-nutrient soil increased phenylpropanoid content in the cucumbers, while most metabolites decreased. In conclusion, fertilization levels should be carefully determined, considering culture conditions such as the original soil status, to increase product yield and fruit quality and avoid environmental problems.

## 1. Introduction

Cucumber (*Cucumis sativus*) is a low-calorie, high-nutrient (vitamins and minerals) vegetable. It is known to have antioxidant, anti-inflammatory, and anticancer effects, which may result from nutrients such as cucurbitacin, lignans, flavonoids, and chlorophylls [1,2,3]. Due to their health benefits, there is a high demand for cucumbers. Thus, farmers have attempted to improve crop yield and quality by applying fertilizers.

Organic fertilizers (e.g., compost and manure) and chemical/industrial fertilizers (e.g., ammonium phosphate and urea) are generally used in agriculture to increase crop yield. However, this has become an issue because the overuse of fertilizers causes many side effects such as environmental pollution. Currently, NPK fertilizer, which is composed of nitrogen, phosphorus, and potassium, is widely used as a chemical fertilizer. A previous study reported that less than half of the supplied fertilizer is used by plants, and the remainder is lost to the environment [4]. The remaining nutrients are washed out of the soil and discharged into waterways (e.g., streams, rivers, and seas), causing various environmental problems. Eutrophication, caused by excess nutrients originating from fertilizers, leads to harmful algal blooms in waterways. In addition, the nitrogen in fertilizer can be converted to N_2_O by soil microorganisms; N_2_O, which has a global warming potential approximately 300 times greater than that of CO_2_, is then released into the atmosphere. Moreover, the overuse of fertilizer results in nutrient leaching and an accumulation of surplus salts in soil that deteriorates the agricultural environment, preventing sustainable agriculture [5,6]. It was reported that soil salinization due to overfertilization is affecting 20% of irrigated land [7]. Excessive amounts of fertilizer not only cause environmental pollution but also negatively affect plants. Overfertilization leads to reduced root growth, shoot size, and premature fruit fall, eventually reducing fruit yield [8,9]. For example, N overfertilization reduces crop yield, particularly for fruit trees and fruit vegetables, including cucumbers, because of a reduction in flower bud initiation and fruit set [10]. Wang et al. (2020) [2] reported that cucumber yield decreased by 40% as a result of severely excessive P supply (3 times the control). Plants exposed to excess nitrogen are more vulnerable to insect, disease, or pathogen attacks [11,12,13]. Moreover, overfertilization also negatively affects internal and external fruit qualities. For example, high N application caused light fruit skin color along with reduced color-relevant metabolite contents [14]. Many researchers have discussed the effects of overfertilization on the environment and plant growth, but only a handful of studies have been performed on the nutritional changes in crops due to the misuse of fertilizers. Even though researchers studied the negative effects of excess fertilizer treatment on fruits, the NPK levels that hinder fruit growth and quality have been inconsistent. Thus, we assumed that differences in the original soil nutrients in each study might have led to inconsistent results. Therefore, to comprehend the differences between previous studies, it is necessary to treat nutritionally different soils with fertilizer.

Most nutritional studies have been limited to analyses of target metabolites, such as carbohydrates, amino acids, and organic acids, which are mainly relevant to taste, or certain metabolites with known bioactive functions [15,16,17]. Recently, advanced high-resolution mass spectrometry (e.g., gas chromatography–mass spectrometry and ultra-performance liquid chromatography–tandem mass spectrometry) and metabolomic approaches have been used to widen the understanding of metabolite composition in foods and plants through untargeted metabolomics [18,19,20]. This helps to analyze a variety of small molecules (≤1500 Da), including primary and secondary metabolites, relevant to the taste, aroma, color, and nutritional qualities of food [21]. Based on the information derived from plant metabolomics, cultivation strategies can be designed to develop fruits and vegetables with desired traits, since the plant metabolome is changed by stresses and culture conditions [22].

In this study, to understand the effects of fertilizer on cucumber fruits, we cultivated cucumbers by treating two chemically different soils with various levels of chemical fertilizer, because the capacity of field soil is diverse. We analyzed cucumber fruits cultivated under different soil conditions and fertilizer levels using a mass-spectrometry-based non-targeted metabolomics approach. To decipher the metabolic differences in cucumber fruits, the different metabolite contents of cucumbers raised in different environments were described on a metabolic pathway map. This study included the following:

- Evaluating the effects of original soil conditions on cucumber fruit yield and quality;
- Assessment of the effects of applied NPK fertilizer levels on the cucumber metabolome in low- and high-nutrient soils;
- Suggesting the necessity for nutrient management based on our results.

## 2. Materials and Methods

### 2.1. Experimental Design and Treatments

We cultivated cucumbers during the spring of 2021 in two plastic film houses: one with low-nutrient soil [optimal phosphorus (Av. P_2_O_5_) but exchangeable K and NO_3_-N were lower than optimal for raising cucumber] and one with high-nutrient soil (high Av. P_2_O_5_ and K, but NO_3_-N was lower than optimal for raising cucumber) (Table 1). The information on recommended optimal soil chemical properties provided by the National Institute of Agricultural Sciences in Korea for cucumber cultivation in plastic houses was used to evaluate the soil properties [18]. The plastic house with low-nutrient soil was in Pyeongtaek, Republic of Korea (37.09305 N, 127.08302 E), and the one with high-nutrient soil was in Cheonan, Republic of Korea (36.74823 N, 127.29853 E). According to the information from the World Reference Base for Soil Resources 2014 [23], low- and high-nutrient soils were classified as Fluvic Gleysols (Siltic, Aric, Drainic, Humic) and Fluvic Gleysoils (Siltic, Drainic, Ochric), respectively. To determine the effect of fertilizer on cucumber fruits in nutritionally different soils, four different concentrations of N-P-K fertilizer [F0, F0.5, F1, and F2] were supplied weekly by fertigation, where F1 was 164:114:131 kg ha^−1^ of N:P_2_O_5_:K_2_O during the cultivation period with a weekly supply amount, as described in Table S1 [24]. Fertilizer was composed of urea (N 46%; Namhae Chemical Corporation, Seoul, Republic of Korea), monopotassium phosphate (P_2_O_5_ 52%, K_2_O 34%; Haifa Group, Haifa, Israel), and potassium sulfate (K 50%; Pungnong, Seoul, Republic of Korea). The weekly fertilizer supply amount of F1 was the standard amount using a fertigation system for cucumbers following the plant nutrient requirement that is different depending on the growth stage, i.e., growth-stage-based fertigation. F0 was no fertilizer treatment, and F0.5 and F2 were 50% and 200% of the F1 amount, respectively. Overall, the eight treatments established were low-nutrient soil treated with F0 (LF0), low-nutrient soil treated with F0.5 (LF0.5), low-nutrient soil treated with F1 (LF1), low-nutrient soil treated with F2 (LF2), high-nutrient soil treated with F0 (HF0), high-nutrient soil treated with F0.5 (HF0.5), high-nutrient soil treated with F1 (HF1), and high-nutrient soil treated with F2 (HF2).

The plastic film house with initially low-nutrient soil was divided into 10 plots (three replicates for LF0.5, LF1, and LF2 and one plot for LF0) to grow cucumber plants under different fertilizer supplies. The cucumber seedlings were planted on 16 March 2021. Each plot contained 80 plants. The harvest was on 24 May 2021 (69 days after planting), and the fruits were ca. 0.25 m long, a length that is proper for purchase in the market. Two fruits out of two different plants of each plot (LF0.5, LF1, and LF2; in total, six fruits out of six different plants for each treatment) and six LF0 fruits out of six different plants were collected for metabolome analysis (i.e., six biological replicates for each treatment).

The plastic film house with initially high-nutrient soil was divided into 13 plots (four replicates for HF0.5, HF1, and HF2 and one plot for HF0) to grow cucumber plants under different fertilizer supplies. The cucumber seedlings were planted on 5 February 2021. Each plot contained 56 plants. Approximately 0.25 m long fruits were harvested on 20 April 2021 (74 d after planting). Two fruits out of two different plants from each plot (HF0.5, HF1, and HF2; in total, eight fruits out of eight different plants for each treatment; we used a higher number of replicates for high-nutrient soil cultivation to compensate for the non-uniform water drainage pattern depending on positions) and eight HF0 fruits out of eight different plants were collected for metabolome analysis (i.e., eight biological replicates for each treatment).

### 2.2. Soil Chemical Property Analysis for Two Different Plastic Film Houses

To analyze the soil chemical properties before planting, three soil samples were collected with an auger at 15 cm depth from each plastic film house. Before analysis, the samples were put together and thoroughly mixed to form composites. All soil parameters were analyzed using the standard analysis methods from the National Institute of Agricultural Sciences as described in a previous study [18]. Soil pH and electrical conductivity (EC) were measured with a pH meter and an EC meter by diluting the soil to a 1:5 ratio of soil to distilled water. The measured EC values were multiplied by a dilution factor of 5. Soil NO_3_-N content was analyzed at 660 nm wavelength by Auto analyzer 3 (QuAAtro, Bran+Luebbe, Norderstedt, Germany). Soil carbon concentrations were determined by the Tyurin method and then converted to soil organic matter (SOM) values by multiplying them by a factor of 1.75. The soil available phosphorus (Av. P_2_O_5_) was extracted by the Lancaster method, and its concentration was calorimetrically quantified at 720 nm wavelength with a UV spectrometer (Hitachi, Tokyo, Japan). Exchangeable cations (Ca, K, and Mg) in the soil were measured with inductively coupled plasma (ICP-OES; Integra XL, GBC, Braeside, Australia) after extraction with 1 N mono-ammonium acetate.

### 2.3. Sample Preparation for Metabolome Analysis

The harvested cucumber fruits were stored at −80 °C. Approximately 20 g of frozen cucumber was placed in a plastic bag and broken into small pieces using a hammer. The broken cucumbers were then lyophilized and ground using a mortar and pestle. The powdered samples were stored at −80 °C until metabolite extraction. The powder was then extracted with 80% methanol (*v*/*v*). Samples were homogenized using a Retsch MM 400 Mixer Mill (Retsch GmbH Co., Haan, Germany) at a frequency of 30 s^−1^ for 10 min and then sonicated for 10 min (Hettich Zentrifugen Universal 320, Tuttlingen, Germany). The samples were centrifuged at 15,000 rpm and 4 °C for 10 min (Gyrozen 1730R, Daejeon, Republic of Korea), and the supernatants were filtered by a polytetrafluoroethylene filter (Chromodisc, Daegu, Republic of Korea). The filtered supernatant was dried overnight in a speed vacuum concentrator (Biotron, Seoul, Republic of Korea). The dried samples were re-dissolved in methanol to a concentration of 10,000 ppm.

### 2.4. Instrumental Analysis

The extracted metabolites were analyzed by gas chromatography time-of-flight mass spectrometry (GC-TOF-MS) and ultrahigh-performance liquid chromatography–linear trap quadrupole Orbitrap mass spectrometry (UHPLC-LTQ-Orbitrap-MS/MS). For GC-TOF-MS analysis, 50 μL of methoxyamine hydrochloride (20 mg/mL in pyridine) was added to the dried extract and incubated at 30 °C for 90 min. After that, 50 μL of MSTFA was added to the reaction mixture and incubated at 37 °C for 30 min. All samples were filtered before instrumental analysis. The GC-TOF-MS analysis was conducted using an Agilent 7890A (Agilent Technologies, Palo Alto, CA, USA) coupled with an Agilent 7693 autosampler and a Pegasus HT TOF-MS (Leco Corporation, St. Joseph, MI, USA). For chromatographic separation, an Rtx-5MS column (30 m length × 0.25 mm inner diameter; J&W Scientific, Santa Clara, CA, USA) was used with helium as the carrier gas at a constant flow (1.5 mL/min). The operational parameters for the analysis were determined as previously reported [25].

A UHPLC system was equipped with a Vanquish binary pump H system (Thermo Fisher Scientific, Waltham, MA, USA) coupled with an autosampler. A Phenomenex KINTEX^®^ C18 column (100 mm × 2.1 mm, 1.7 μm particle size; Torrance, CA, USA) was used for chromatographic separation. The column temperature and flow rate were maintained at 40 °C and 0.3 mL/min, respectively. MS data were collected within the range of 100–1000 m/z in both negative and positive ion modes using an Orbitrap Velos Pro^TM^ system combined with an ion-trap mass spectrometer (Thermo Fisher Scientific) coupled with a HESI-II probe. The analytical methods were adopted from a previous study [26].

### 2.5. Data Processing and Multivariate Statistical Analysis

The raw data obtained from GC-TOF-MS were converted into the Net CDF format using LECO Chroma TOF software (LECO Corporation). The raw UHPLC-LTQ-Orbitrap-MS/MS data were converted to the Net CDF format using Xcalibur software (Thermo Fisher Scientific). The converted CDF files were preprocessed using MetAlign software for alignment based on peak detection and retention time correction. SIMCA-P+ software (Umetrics, Umeå, Sweden) was used for multivariate statistical analysis. Significantly different metabolites were selected by variable importance in projection (VIP > 1.0) values based on partial least squares discriminant analysis (PLS-DA) score plots and a significance test (*p*-value < 0.05) by analysis of variance (ANOVA) using the STATISTICA 7 software (StatSoft, Tulsa, OK, USA). The selected compounds were tentatively identified by comparing their retention times, mass fragment patterns, and elemental compositions with those of standard compounds, published data, and/or public databases such as the National Institute of Standards and Technology (NIST) Library (version 2.0, FairCom, Gaithersburg, MD, USA), Wiley8, and the Human Metabolome Database (HMDB; http://www.hmdb.ca/).

## 3. Results

### 3.1. Yields of Cucumber Fruits Cultivated in Two Nutritionally Different Soils

To compare the yields under different growing conditions, marketable cucumbers (ca. 0.25 m in length) were harvested and weighed. From the low-nutrient soil, the yield of cucumber harvested from 16 March 2021 to 26 July 2021 (132 days) ranged from 98 to 105 tons ha^−1^ with no statistical differences among F0.5, F1, and F2 (Table S2). Similarly, the yield of cucumbers from the high-nutrient soil ranged from 119 to 122 tons ha^−1^ with no statistical differences among F0.5, F1, and F2; these cucumbers were collected from 5 February 2021 to 28 June 2021 (143 days) (Table S2). This implies that fertilizer supply does not significantly affect fruit yield under low- and high-nutrient soil conditions. Additionally, lower partial factor productivity (PFP), the ratio of total output to total inputs, was shown with a higher fertilizer supply in both nutritionally different soils. This means that fertilizer use efficiency was low when more fertilizer was supplied.

### 3.2. Soil Chemical Properties of Two Nutritionally Different Soils at Fruit Harvesting

To compare the soil chemical properties and the fruit metabolites, fruit samples and soil samples were collected on the same day. The average values of soil chemical properties except pH were statistically higher in the high-nutrient soil condition than in low-nutrient soil (Table 2). The different NPK fertilizer levels (F0.5, F1, and F2) insignificantly affected soil chemical properties in both low- and high-nutrient soils (ANOVA, *p*-value > 0.05).

### 3.3. Non-Targeted Metabolite Profiling and Metabolism Comparison of Cucumber Fruits Cultivated in Two Nutritionally Different Soils

**Figure 1:**
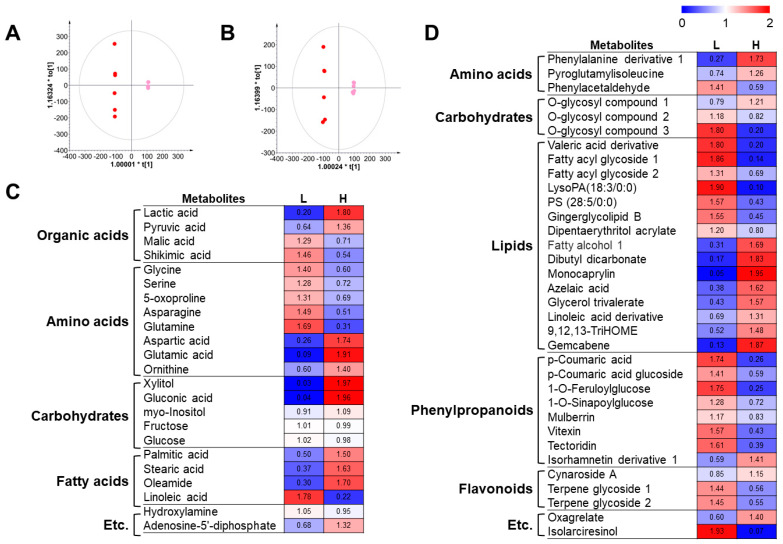
Orthogonal projection to latent structures discriminant analysis (OPLS-DA) (**A**,**B**) for metabolites in cucumbers grown in two different soil conditions based on GC-TOF-MS (**A**) and UHPLC-LTQ-Orbitrap-MS/MS (**B**) data set. Heatmap analysis of cucumbers from two different soils based on GC-TOF-MS (**C**) and UHPLC-LTQ-Orbitrap-MS/MS (**D**) data. Heatmap represents the relative abundance of significantly discriminant metabolites (VIP > 1.0, *p*-value < 0.05) based on the OPLS-DA model and one-way ANOVA test. Cucumbers from low-nutrient soil (●) and cucumbers from high-nutrient soil (●).

To examine the metabolite differences in cucumber fruits cultivated in nutritionally different soils, we performed metabolite profiling using GC-TOF-MS and UHPLC-LTQ-Orbitrap-MS/MS analysis results. The OPLS-DA score plots derived from both instrumental analyses showed a clear distinction between the low- and high-nutrient soils (Figure 1A,B). We selected and identified significantly discriminant metabolites in cucumber fruits grown in two nutritionally different soils based on the VIP value (>1.0) and *p*-value (<0.05) based on OPLS-DA and one-way ANOVA, respectively.

A total of 57 metabolites were identified, 23 and 34 of which were analyzed using GC-TOF-MS and UHPLC-LTQ-Orbitrap-MS/MS, respectively (Figure 1C,D). The identified metabolites included 11 amino acids, 4 organic acids, 8 carbohydrates, 19 lipids, 8 phenylpropanoids, and 3 flavonoids. Similar to the OPLS-DA score plots, the heat map showed a clear distinction between cucumbers harvested from low- and high-nutrient soils. The cucumbers harvested from low-nutrient soil contained higher amounts of most amino acids, phenylpropanoids, and some lipids than the fruits raised in high-nutrient soil. Interestingly, lactic acid, pyruvic acid, aspartic acid, glutamic acid, and ornithine were comparatively more abundant in cucumbers from high-nutrient soil. In addition, the lipid composition in cucumbers from high-nutrient soil is obviously different as compared to the fruit from low-nutrient soil.

**Figure 2:**
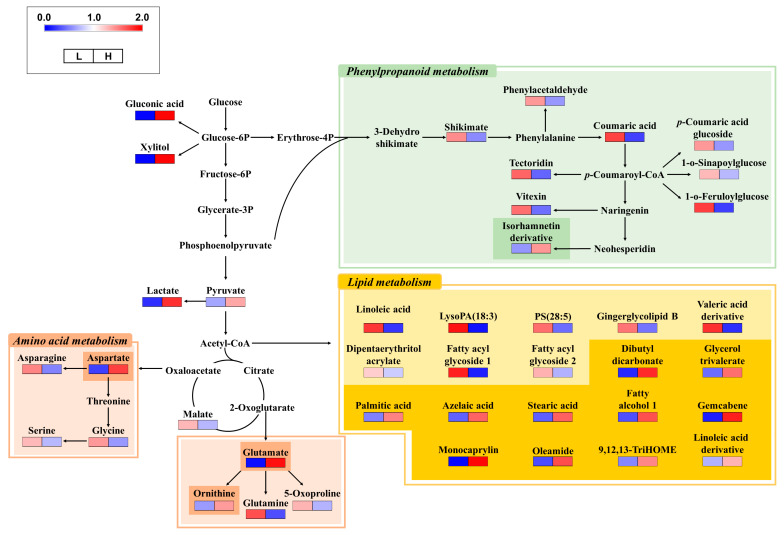
Metabolic pathways and relative levels of metabolites in cucumbers from two different soils. The relative levels of metabolites are represented as fold changes normalized using the average of all values. The metabolic pathway was modified based on the KEGG database (https://www.genome.jp/kegg/). L, low-nutrient soil; H, high-nutrient soil.

To visualize the metabolic differences between the cucumbers from the two nutritionally different soils, we reconstructed the metabolic pathway maps of cucumbers based on the identified metabolites (Figure 2). The relative levels of each metabolite are represented as a heat map in the biosynthetic pathways. Numerous amino acids were abundant in cucumbers from the low-nutrient soil (light orange box in Figure 2), excluding aspartate, glutamate, and ornithine (dark orange box in Figure 2), derived from the tricarboxylic acid cycle and the metabolic branches for synthesizing other amino acids. Most phenylpropanoids (light green box in Figure 2) were higher in cucumbers grown in low-nutrient soil, except for one phenylpropanoid (isorhamnetin derivative; dark green box in Figure 2), which was the most distal from the precursor (coumaric acid). Some lipids were relatively abundant in cucumbers from low-nutrient soil (light yellow in Figure 2), whereas others were more abundant (dark yellow in Figure 2).

### 3.4. Effect of NPK Fertilizers on Metabolome of Cucumber Fruits in the Two Different Soils

Next, we investigated the effect of NPK fertilizer concentration on cucumber metabolomics in each soil type.

**Figure 3:**
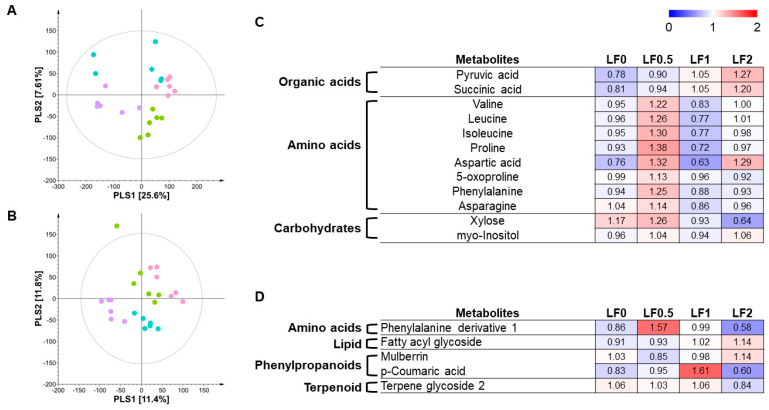
Partial least squares discriminant analysis (PLS-DA) (**A**,**B**) of metabolites in cucumbers treated with different levels of fertilizer in low-nutrient soil conditions based on GC-TOF-MS (**A**) and UHPLC-LTQ-Orbitrap-MS/MS (**B**) data set. Heatmap analysis of cucumbers treated with different levels of fertilizer in low-nutrient soil based on GC-TOF-MS (**C**) and UHPLC-LTQ-Orbitrap-MS/MS (**D**) data. Heatmap represents the relative abundance of significantly discriminant metabolites (VIP > 1.0, *p*-value < 0.05) based on the PLS-DA models and one-way ANOVA tests. Cucumbers treated with F0 in low-nutrient soil (●), cucumbers treated with F0.5 in low-nutrient soil (●), cucumbers treated with F1 in low-nutrient soil (●), and cucumbers treated with F2 in low-nutrient soil (●).

In the case of the low-nutrient soil condition, the PLS-DA score plots using GC-TOF-MS and UHPLC-LTQ-Orbitrap-MS/MS showed a shift depending on the fertilizer level (Figure 3A,B). According to the multivariate statistical analysis results, we selected 12 and 5 significantly different metabolites from the GC-TOF-MS and UHPLC-LTQ-Orbitrap-MS/MS analysis data, respectively (Figure 3C,D). The identified metabolites included nine amino acids, two organic acids, two carbohydrates, one lipid, two phenylpropanoids, and one terpenoid. Unexpectedly, the significantly different metabolites did not show a changing pattern with increasing fertilizer levels. Amino acids were the most abundant in cucumbers treated with F 0.5, whereas organic acids were the most abundant in the F2 treatment.

**Figure 4:**
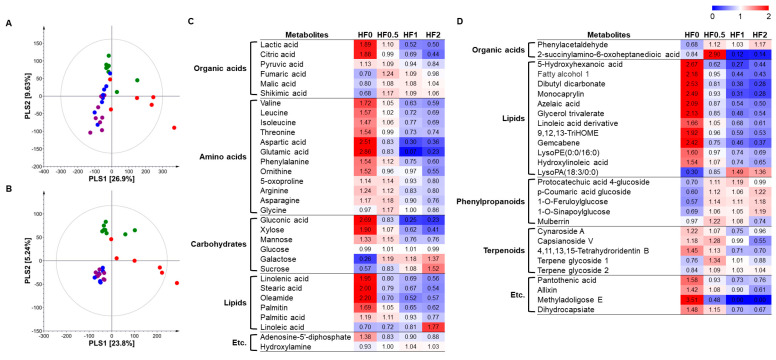
Partial least squares discriminant analysis (PLS-DA) (**A**,**B**) of metabolites in cucumbers treated with different levels of fertilizer in high-nutrient soil conditions based on GC-TOF-MS (**A**) and UHPLC-LTQ-Orbitrap-MS/MS (**B**) data set. Heatmap analysis of cucumbers treated with different levels of fertilizer in high-nutrient soil based on GC-TOF-MS (**C**) and UHPLC-LTQ-Orbitrap-MS/MS (**D**) data. Heatmap represents the relative abundance of significantly discriminant metabolites (VIP > 1.0, *p*-value < 0.05) based on the PLS-DA models and one-way ANOVA tests. Cucumbers treated with F0 in high-nutrient soil (●), cucumbers treated with F0.5 in high-nutrient soil (●), cucumbers treated with F1 in high-nutrient soil (●), and cucumbers treated with F2 in high-nutrient soil (●).

The PLS-DA score plots derived from both instrumental analyses showed a shifting pattern from F0 to F2 under high-nutrient soil conditions (Figure 4A,B). Based on the VIP value (>1.0) obtained using PLS-DA and *p*-value (<0.05) from one-way ANOVA, we selected and identified significantly different metabolites in the cucumber fruits. A total of 60 metabolites were identified as significantly different, of which 32 and 28 were analyzed using GC-TOF-MS and UHPLC-LTQ-Orbitrap-MS/MS, respectively (Figure 4C,D). The identified metabolites included 12 amino acids, 8 organic acids, 6 carbohydrates, 18 lipids, 5 phenylpropanoids, and 5 terpenoids. The heat map shows the altered metabolites were affected by different NPK fertilizer levels in cucumber fruits. The amounts of amino acids, lipids, and terpenoids decreased, whereas those of some organic acids (fumaric acid, malic acid, and shikimic acid), carbohydrates (galactose and sucrose), and phenylpropanoids increased with higher fertilizer levels.

## 4. Discussion

The overuse of fertilizers has become an issue because of environmental problems such as eutrophication and global warming [27,28]. Moreover, overfertilization also negatively affects crop yield, fruit quality, and metabolite contents in fruits and vegetables [2,10,14,29]. Therefore, to investigate the effective fertilizer levels that can improve fruit quality with fewer environmental problems, we raised cucumbers by treating two nutritionally different soils with four different levels of fertilizer, and then, we evaluated the yield, quality of cucumber fruit, and soil chemical properties at the harvest time. The fruit quality was assessed using metabolomic approaches.

As expected, the metabolite composition of cucumbers differed remarkably according to the original soil nutrient conditions, even when various concentrations of NPK fertilizers were used. Cucumbers raised in high-nutrient soil without fertilizer contained more glutamic acid and aspartic acid, contributing to the umami taste of the food, compared to other conditions. In contrast, other amino acids, such as glycine, serine, and asparagine, which elicit a sweet–bitter taste, were abundant in cucumbers from low-nutrient soil, implying that growing soil conditions affect the taste of food [30]. In cucumbers raised in low-nutrient soil, the contents of most phenylpropanoids were higher than those in the fruit from the high-nutrient soil, excluding an isorhamnetin derivative. In cucumbers harvested from the low-nutrient soil with NPK fertilizer treatment, the contents of phenylpropanoids (e.g., mulberrin and *p*-coumaric acid) were perturbed by fertilizer treatment, similar to other metabolites. Unlike what was observed in the low-nutrient soil condition, increased levels of shikimic acid and phenylpropanoids, such as *p*-coumaric acid glucoside, mulberrin, and 1-O-feruloylglucose, which possess antioxidant activities, were observed in cucumbers cultivated in high-nutrient soil with higher levels of fertilizer [31,32,33]. However, the increase in phenylpropanoids was not significant as compared to the decrease in other nutrients, including organic acids, amino acids, and lipids. Taken together, these results indicate that the metabolite contents in fruits are mainly affected by the traits of the original soil rather than the fertilizer treatment. Even though the fertilizer supply can change the metabolites in fruits, the alteration can be nutritionally negative. Thus, to improve fruit yield and quality, careful fertilizer application design must be conducted after an assessment of the original soil properties.

In the current study, phenylpropanoids were among the major metabolites in cucumber that were changed by original soils and fertilizer treatment. It is well known that the synthesis of phenylpropanoids can be induced by various stresses. For example, abiotic stresses (e.g., drought, salinity, UV radiation, heavy metal toxicity) can cause physiological changes, resulting in plant growth inhibition and yield reduction [34]. Moreover, they can alter the metabolite composition in plants, leading to enhanced accumulation of secondary metabolites (e.g., phenols, flavonoids, anthocyanins), eventually protecting plants from stressful environments [34]. Phenylpropanoids are synthesized from aromatic amino acids (e.g., phenylalanine and tyrosine) via phenylalanine ammonia-lyase (PAL), which is a key enzyme for phenylpropanoid biosynthesis in plants, as described in Figure 2 [34,35]. In this study, phenylpropanoids were increased with the application of higher concentrations of fertilizer in the high-nutrient soil condition, implying the fertilizer might act as an abiotic stressor. On the other hand, applying different levels of fertilizer in the low-nutrient soil condition insignificantly affected fruit yield and metabolites, including phenylpropanoids. We assumed that other factors (apart from the NPK levels) might cause cucumber fruits to contain relatively abundant phenylpropanoids and were not significantly changed by the fertilizer treatment.

The soil chemical properties at the fruit harvest time were statistically different between the low- and high-nutrient soil conditions. Notably, the fertilizer supply levels did not significantly affect the soil chemical properties. This may indicate that nutrient leaching into the environment could be higher in the case of a higher fertilizer supply, considering the yield and plant nutrient uptake. Based on numerous studies, including this one, it is necessary to implement nutrient management to optimize nutrient use efficiency without causing environmental pollution. To establish a nutrient management plan, soil tests (e.g., pH, N-P-K, and other chemical properties) must be conducted, and nutrient supply methods (e.g., fertigation) and rates should be appropriately designed [36,37,38,39].

## 5. Conclusions

Fertilizers are generally used to improve the productivity and quality of fruits and vegetables. Recently, the overuse of fertilizers has become an issue because it can cause environmental pollution. Thus, it is important to use appropriate levels of fertilizers to reduce environmental problems while increasing fruit yield and quality. Therefore, in the present study, we evaluated the yield and quality of fruits by performing non-targeted metabolite profiling of cucumbers raised in two nutritionally different soils treated with different fertilizer levels. We observed that cucumbers grown in low-nutrient soil were relatively abundant with most amino acids, phenylpropanoids, and some lipids, whereas the levels of some carbohydrates, glutamic acid, and aspartic acid were higher in cucumbers from high-nutrient soil. The fruit yield was not significantly changed in both low- and high-nutrient soil conditions by the fertilizer treatment, while the metabolite profiling was changed. The fertilizer supply perturbed the metabolites in cucumbers from low-nutrient soil. By contrast, phenylpropanoids were increased with a higher amount of fertilizer, whereas most metabolites decreased in cucumbers grown in high-nutrient soil. As a result, the fruit yield and metabolome of cucumber fruits were affected more by the original soil than by fertilizer levels. Moreover, the fertilizer treatment negatively affected fruit metabolite contents under specific conditions. Therefore, fertilizer treatment levels should be carefully designed based on an assessment of the original soil status.

## Figures and Tables

**Table 1:** Concentration of nitrate nitrogen, available phosphorus pentoxide, and exchangeable potassium in the two different original soils before planting.

Chemical Properties	NO_3_-N (mg kg^−1^)	Av. P_2_O_5_ (mg kg^−1^)	K (cmol_c_ kg^−1^)
Low-nutrient soil	32	432	0.41
High-nutrient soil	53	1147	0.85
Recommended *	70–200	350–450	0.65–0.80

Mean values of triplicate samplings. * The optimal values for cucumber cultivation in Korean plastic house.

**Table 2:** Chemical properties of two different soils at the fruit sample harvest for the metabolite profiling.

Chemical Properties	pH (1:5)	EC (1:5; dSm^−1^)	NO_3_-N (mg kg^−1^)	OM (g kg^−1^)	Av. P_2_O_5_ (mg kg^−1^)	Ex. Cations (cmol_c_/kg^−1^)			
						K	Ca	Mg	Na
Low-nutrient soil	6.2 ^a^	1.8 ^b^	45 ^b^	22 ^b^	428 ^b^	0.91 ^b^	6.7 ^b^	2.2 ^b^	0.23 ^b^
High-nutrient soil	6.4 ^a^	4.5 ^a^	99 ^a^	49 ^a^	1525 ^a^	1.88 ^a^	12.5 ^a^	2.9 ^a^	0.74 ^a^

Mean values of 10 soil samples from low-nutrient soil and 13 soil samples from high-nutrient soil. Different alphabetical letters in a column indicate significant differences, as determined by unpaired Student’s *t*-test (*p*-value < 0.05).

## Data Availability

The data presented in this study are available in the article and Supplementary Materials.

## Supplementary Materials

The following supporting information can be downloaded at https://www.mdpi.com/article/10.3390/metabo14020102/s1: Table S1: Weekly fertilizer supply (kg ha^−1^), Table S2: Cucumber yield and partial factor productivity (PFP).
