# Supplementary material for: Metabolomics Reveals the Effects of Nitrogen/Phosphorus/Potassium (NPK) Fertilizer Levels on Cucumber Fruit Raised in Different Nutrient Soils
Source: Metabolites. 2024 Feb 1;14(2):102. doi: 10.3390/metabo14020102 (PMC10891504; doi:10.3390/metabo14020102)
Supplement: Supplementary file 1 [file metabolites-14-00102-s001.zip › metabolites-2800825-supplementary.pdf]

Table S1. Weekly fertilizer supply of different fertilizer levels

Weeks after transplanting	F0.5 (N-P-K; kg ha^{-1})	F1 (N-P-K; kg ha^{-1})	F2 (N-P-K; kg ha^{-1})
3−4	3-2-2	6-4-5	12-8-9
5−12	6.5-5-5	13-9-11	26-18-21
13−21	6-4-5	12-8-9	24-16-19
**Total**	**82-57-65**	**164-114-131**	**328-228-262**

Table S2. Cucumber yield and Partial Factor Productivity (PFP)

Low-nutrient soil	Fertilizer supply (kg ha^{-1})	Yield† (kg ha^{-1})	PFP‡ (kg kg^{-1})
F0	0	100,600	-
F0.5	205	104,500	510
F1	409	98,100	240
F2	819	103,700	127

High-nutrient soil	Fertilizer supply (kg ha^{-1})	Yield† (kg ha^{-1})	PFP‡ (kg kg^{-1})
F0	0	119,000	-
F0.5	205	122,000	596
F1	409	119,000	291
F2	819	122,000	149

†There was no statistical difference in the yield by the different fertilizer supply from F0.5 to F2 (ANOVA test, *p*-value > 0.05).

‡PFP (Partial Factor Productivity) = Yield/Fertilizer supply (N+P+K).
